# Energy Storage Characteristics of BiFeO_3_/BaTiO_3_ Bi-Layers Integrated on Si

**DOI:** 10.3390/ma9110935

**Published:** 2016-11-18

**Authors:** Menglin Liu, Hanfei Zhu, Yunxiang Zhang, Caihong Xue, Jun Ouyang

**Affiliations:** 1 Key Laboratory for Liquid−Solid Structural Evolution and Processing of Materials (Ministry of Education), School of Materials Science and Engineering, Shandong University, Jinan 250061, China; liumenglinnadou@163.com (M.L.); hfzhu1986@163.com (H.Z.); zhangyunxiangls@163.com (Y.Z.); chxueaj@163.com (C.X.); 2 Suzhou Institute of Shandong University, Suzhou 215123, China; 3 National Institute of Standards and Technology, Gaithersburg, MD 20899, USA

**Keywords:** ferroelectrics, lead-free, energy storage, bilayer, BiFeO_3_, BaTiO_3_, Si

## Abstract

BiFeO_3_/BaTiO_3_ bi-layer thick films (~1 μm) were deposited on Pt/Ti/SiO_2_/(100) Si substrates with LaNiO_3_ buffer layers at 500 °C via a rf magnetron sputtering process. X-ray diffraction (XRD) analysis revealed that both BiFeO_3_ and BaTiO_3_ layers have a (00*l*) preferred orientation. The films showed a small remnant polarization (*P_r_* ~ 7.8 μC/cm^2^) and a large saturated polarization (*Ps* ~ 65 μC/cm^2^), resulting in a slim polarization-electric field (*P-E*) hysteresis loop with improved energy storage characteristics (*W_c_* = 71 J/cm^3^, *η* = 61%). The successful “slim-down” of the *P-E* loop from that of the pure BiFeO_3_ film can be attributed to the competing effects of space charges and the interlayer charge coupling on charge transport of the bi-layer film. The accompanying electrical properties of the bi-layer films were measured and the results confirmed their good quality.

## 1. Introduction

Densities of electrical energy stored in or released from a dielectric can be calculated from their characteristic dielectric displacement-electric field (*D-E*) curves by the formula [1]:

(1a) $$W_{c}=\int_{0}^{D_{s}}EdD,W_{d}=\int_{D_{r}}^{D_{s}}EdD$$

(1b) $$\eta =\frac{W_{d}}{W_{c}},$$

where *W_c_* is the charged (stored) energy density, *W_d_* is the discharged (released) energy density and *η* the energy efficiency. *D_s_* and *D_r_* are the saturated/maximum and remnant electrical displacements, respectively. For an ideal linear dielectric with a relative dielectric constant *ε_r_*, *D* = *ε*_0_*ε_r_E* and *D_r_* = 0, the energy densities are given by [1]:

(2a) $$W_{c}=\frac{1}{2}\varepsilon_{0}\varepsilon_{r}E_{\max}^{2},$$

(2b) $$W_{d}=W_{c}=\frac{1}{2}\varepsilon_{0}\varepsilon_{r}E_{\max}^{2},$$

and the efficiency *η* equals 1. Here *ε*_0_ is the vacuum dielectric constant, *E_max_* is the maximum applicable electric field which increases with the breakdown field *E_b_* (*E_max_* ≈ *E_b_* for an ideal dielectric). On the other hand, non-linear dielectrics like ferroelectrics usually don’t have *D* ∝ *E* or *D_r_* = 0, hence *W_c_* > *W_d_* and *η* < 1. Nevertheless, Equation (2a) can still be used for the estimation of energy densities *W_c_* in non-linear dielectrics, wherein *ε_r_* is the average or effective dielectric constant.

**Figure 1:**
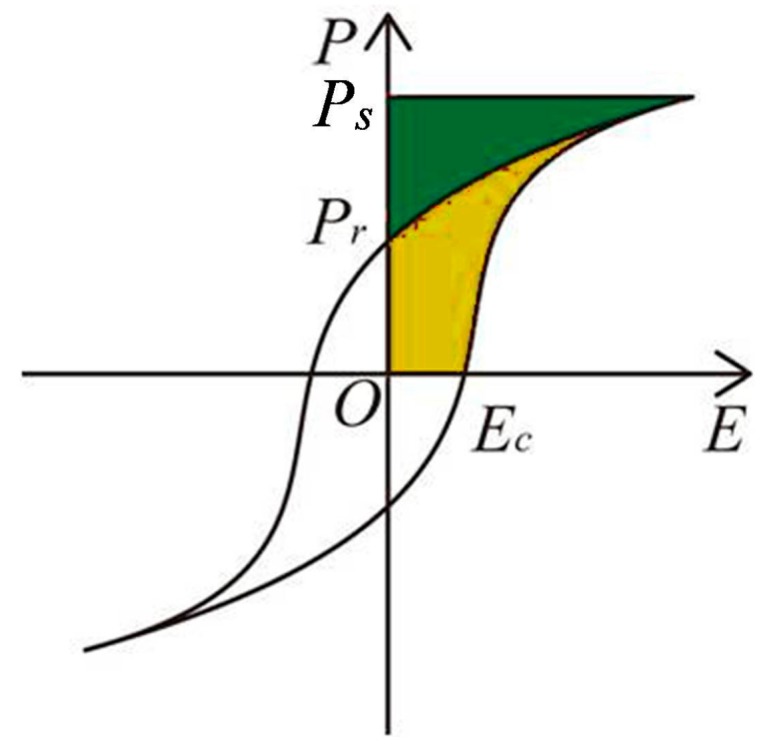
Schematic of a ferroelectric *P-E* loop. The green area is the discharged energy density *W_d_*, while the yellow area is the energy loss of one charge-discharge cycle (*W*_loss_).

To meet the demands in developing portable and integrable power electronics, thin film ceramic capacitors with a high energy storage density *W_c_* and a high efficiency *η* have been intensively investigated [2,3,4]. The previous studies on thin film ceramic capacitors have been focused on lead-containing perovskite ferroelectrics, including BNZ-PT, BNT-PT and BNH-PT [5,6,7]. *P-E* (*) curve of a typical ferroelectric is presented in Figure 1. In this figure, the green area represents the discharged energy density (*W_d_*), the yellow area encircled by the hysteresis loop is the energy loss (*W*_loss_), and the charged energy density is the sum of the two, *W_c_* = *W_d_* + *W*_loss_. It’s obvious that the energy storage characteristics of a ferroelectric strongly depend on the shape of its *P-E* loop.

In the past decade, large energy storage densities (40 J/cm^3^–60 J/cm^3^) have been reported in lead-containing ferroelectric films together with good energy efficiencies (40%–60%) [6,7,8,9]. However, the growing environment concerns on lead and the usually complex compositions of the solid solutions being used are the two major drawbacks of the lead-containing ferroelectric capacitors. In order to minimize the environmental impact and promote a manufacturing-friendly preparation process, single component lead-free perovskites have been investigated by several research groups for applications in thin film dielectric capacitors [10,11,12,13,14]. Among them, BiFeO_3_ and BaTiO_3_ films are most popular candidates [13,14]. The former has a giant saturated polarization and tunable electrical properties via strain or chemical doping, while the latter has excellent dielectric properties (large *ε_r_* and high *E_b_*) and hence has been broadly used in multi-layer ceramic capacitors (MLCC). However, large leakage currents and poor energy efficiencies associated with BiFeO_3_ thin films have hindered its further development for energy storage applications. On the other hand, a small saturated polarization of BaTiO_3_ has capped its energy densities (see Equation (1a)) and limited most of its capacitor applications in MLCC.

According to Equation 1, dielectrics with high *E_b_* and *ε_r_*, a small remnant polarization and a large saturated polarization are ideal for high efficiency energy-storage applications. In this work, we demonstrate a novel approach to compensate for the lack of a single material with the above properties. We prepared ferroelectric bilayers of BiFeO_3_/BaTiO_3_ on Si substrates via an in-situ rf magnetron sputtering process at 500 °C. Textured growth of the bi-layer films at this moderate temperature was promoted by using a LaNiO_3_ template layer. The resulted thin film capacitors of Au/BiFeO_3_/BaTiO_3_/LaNiO_3_/Pt/Ti not only showed good dielectric properties (high *ε_r_* and low leakage current), but also a slim *P-E* hysteresis featuring an enhanced energy storage density and efficiency, as compared with those of single layers of BiFeO_3_ and BaTiO_3_ on Si. The improved energy storage characteristics are interpreted based on our understanding of the charge transport process, which is dominated by the effect of space charges at low electric fields and by the interface charge coupling at high electric fields.

## 2. Experimental Section

**Figure 2:**
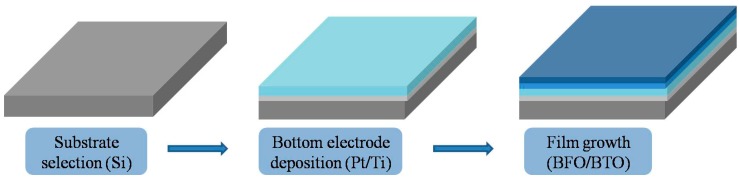
Schematics of the experimental procedures.

BiFeO_3_/BaTiO_3_ bi-layer thick films were fabricated on Pt/Ti/SiO_2_/(100)Si substrates buffered with a LaNiO_3_ layer via a rf magnetron sputtering process. Commercially available ceramic targets of BaTiO_3_, Bi_1.05_FeO_3_ and LaNiO_3_ (4N purity, *Ф* = 50 mm, *L* = 5 mm) were used in the deposition process, which was carried out in a multi-target magnetron sputtering system with a base pressure of 2.0 × 10^−4^ Pa. Firstly, a 120-nm-thick Pt/Ti layer was deposited as the bottom electrode at 300 °C in a pure argon atmosphere. Then the LaNiO_3_, BaTiO_3_ and BiFeO_3_ layers were sequentially sputtered at 500 °C in a mixed gas of Ar and O_2_. The thickness of the BiFeO_3_/BaTiO_3_ bi-layer was about 1 μm with a 1:1 thickness ratio. Lastly, the as-deposited multi-layer thick film was cooled down at a rate of 6 °C/min–8 °C/min in pure oxygen. The deposition parameters of the sputtering process were summarized in Table 1 and the schematics of experimental procedures was shown in Figure 2. For electrical characterizations, circular Au top electrodes with a diameter of 200 μm were deposited at room temperature by using a shadow mask.

The phase structures and crystallographic orientations of the bilayer films were characterized by using X-ray diffraction (XRD) *θ*-2*θ* scans with a Ni-filtered Cu-K*α* radiation resource (Dmax-rc, Monaghan, Ireland) and pole figures (R-156 Axis Spider, SmartLab^®^ Rigaku, Tokyo, Japan, 40 kV, 200 mA). A commercially available MicroNano D-5A Scanning Probe Microscope (SPM) (MicroNano, Shanghai, China) was used to analyze the surface morphology while the cross-sectional thin film morphology was analyzed by using a thermal field emission scanning electron microscope (SEM) (SU-70, HITACHI, Hitachi, Japan) The room temperature ferroelectric hysteresis loops (*P-V*) and leakage currents (*I-V*) were measured by using a Radiant Precision Premium II ferroelectric tester (Radiant Technology, Albuquerque, NM, USA). The dielectric properties were measured by using a high precision digital bridge (QuadTech 7600 Plus Precision LCR Meter, IET LABS, Inc., West Roxbury, MA, USA).

## 3. Results

### 3.1. Microstructures and Crystallographic Orientations

**Figure 3:**
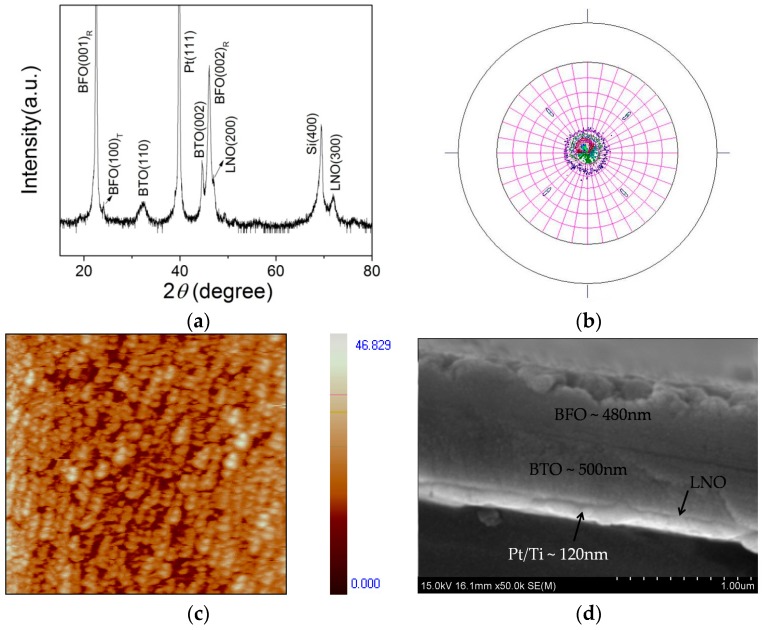
(**a**) XRD 2*θ* scan spectrum (R-rhombohedral, T- tetragonal for BFO peaks, while the BTO phase is tetragonal); (**b**) X-ray pole figure analyses by using the (001)_R_ BFO peak; (**c**) AFM surface scan image (5 μm × 5 μm); and (**d**) cross-sectional SEM image of the BiFeO_3_/BaTiO_3_ bi-layer film deposited on LaNiO_3_/Pt/Ti/SiO_2_/(100) Si substrate.

Figure 3a shows the XRD 2*θ* scan spectrum of the BiFeO_3_/BaTiO_3_ bilayer film grown on LaNiO_3_/Pt/Ti/SiO_2_/(100) Si, which is dominated by the preferred (00*l*) diffraction peaks of the bulk perovskite structures and does not show any crystalline impurities or secondary phases. A tiny amount of (110)-oriented BaTiO_3_ grains and (100)-oriented tetragonal BiFeO_3_ grains were detected, which can be attributed to the moderate growth temperature and the effect of residual stress [15,16]. In our previous work [17], it was revealed that a highly (00*l*)-oriented BaTiO_3_ thin film can be grown on Pt/Ti/SiO_2_/(100) Si substrate by using a LaNiO_3_ buffer layer at 500 °C. Here it is confirmed that the presence of a (*l*00)-oriented LaNiO_3_ buffer layer promoted the growth of a (00*l*) oriented BiFeO_3_/BaTiO_3_ bi-layer film.

To further investigate the crystallographic characteristics of the bi-layer film, pole figure analysis was carried out on the {001} BiFeO_3_ reflection (2*θ* = 22.56°). During collection of the XRD signals, the sample was rotated by varying the tilt angle (0° < *ψ* < 70°) and the azimuthal angle (0° < *φ* < 360°) with respect to the scattering vector. The pole figure of (001)_BFO_, as shown in Figure 3b, revealed a strong diffraction peak at the center (tilt angle *ψ* = 0°), and a set of 4-fold diffraction peaks at *ψ* ≈ 46°, corresponding to the {101} BiFeO_3_ plane. This result confirmed the preferred (00*l*) orientation of the BiFeO_3_ layer, as suggested by the result of the XRD 2*θ* scans.

Surface morphology of the BiFeO_3_/BaTiO_3_ bi-layer film is displayed in Figure 3c. It can be seen that the grains of the top BiFeO_3_ layer are densely packed with an average size of ~200–300 nm. The surface roughness Ra was measured to be ~4.1 nm, which shows a significant reduction as compared with those of the single layer BiFeO_3_ films (Ra is on the order of ~10 nm or above) [18,19,20,21]. The dense and smooth growth of the BiFeO_3_ layer can be attributed to the good quality perovskite underlayers of LaNiO_3_/BaTiO_3_. In Figure 3d, the cross-sectional SEM image shows clean and sharp interfaces in the multi-layer thin film. The BiFeO_3_ and BaTiO_3_ layers were measured to be ~480 nm and ~500 nm, respectively.

### 3.2. Energy Storage Characteristics from the P-E Hysteresis Loop

**Figure 4:**
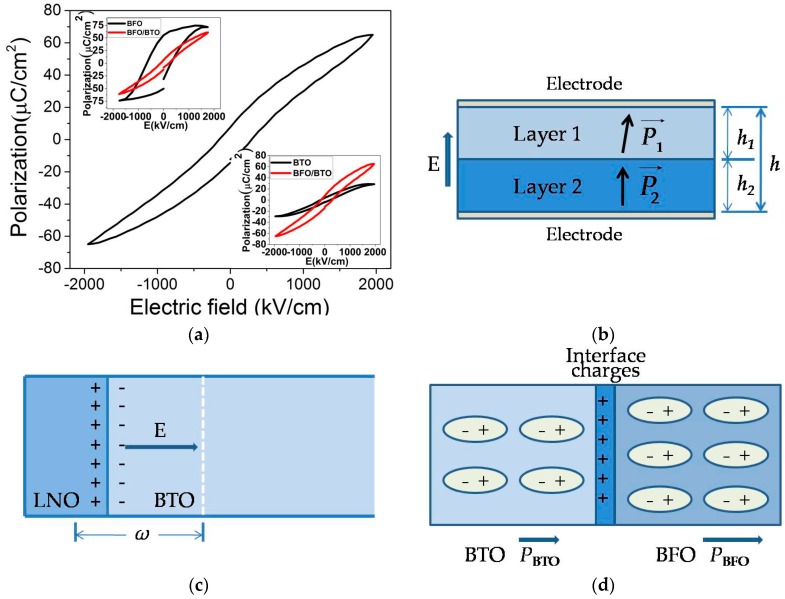
(**a**) Room temperature ferroelectric hysteresis loop of the BiFeO_3_/BaTiO_3_ bi-layer film deposited on LaNiO_3_/Pt/Ti/SiO_2_/(100)Si substrate, the insets compare *P-E* loops of the pure BiFeO_3_ and BaTiO_3_ films with those of the bi-layer film measured under the same electric field [22,23]; (**b**) schematics of the ferroelectric bi-layer considered in our work; (**c**) the space charge effect dominant at a low electric field, ω is the depletion layer width under an electric field E; (**d**) strong interlayer charge coupling dominant at a high electric field (after the BaTiO_3_ layer has been fully depleted).

The poling and depoling process of a dielectric under an external electric field simulates the charge-discharge process of a capacitor. Therefore, from the polarization–electric field (*P-E*) hysteresis loop, the energy storage density *W_c_* and the energy efficiency *η* of the Au/BiFeO_3_/BaTiO_3_/LaNiO_3_/Pt/Ti capacitors can be calculated by using Equation (1). Figure 4a displays the *P-E* hysteresis loop measured at a pseudo-static frequency of 1 kHz for the bi-layer film (under an applied electric field of 1940 kV/cm). A large maximum polarization (*P_m_* ~ 65 μC/cm^2^) and a small remnant polarization (*P_r_* ~ 7.8 μC/cm^2^) are simultaneously obtained, together with a reduced coercive field *E_c_* (*E_c_ ~* 152 kV/cm) as compared with that of pure BiFeO_3_ film [22]. These features of the *P-E* curve ensure excellent energy storage characteristics of the film. When compared with pure BaTiO_3_ films deposited under similar conditions [23], the energy storage density *W_c_* of the bi-layer film reached 71 J/cm^3^ (*E_max_* = 1940 kV/cm), a 100%–110% improvement, while the energy efficiency *η* stayed about the same level (~61% vs. 60%–70%). This improvement in *W_c_* can be attributed to a much improved maximum polarization and a high dielectric strength in par with that of a pure BaTiO_3_ film.

The “slim-down”of the *P-E* loop can be explained by the competition between the space charge effect and interlayer charge coupling in the bi-layer film. As demonstrated in our previous study, a space charge layer in BaTiO_3_ near its interface with LaNiO_3_ dominates the film’s electrical characteristics at a low electric field [24]. Basically, a depletion region with width *ω* forms at the BaTiO_3_/LaNiO_3_ interface upon the application of a small electric field. The width *ω* can be computed by [24]:

(3) $$w=\sqrt{\frac{2\varepsilon (V+V_{bi}^{*})}{qN_{eff}}},$$

where $V_{bi}^{*}$ is the modified built-in voltage, *ε* is the dielectric constant (*ε = ε*_0_*ε_r_*), *N*_eff_ is the space charge concentration, *V* is the applied electric voltage and *q* the electronic charge. The voltage *V* is concentrated across the depletion region and drives its expansion until the BaTiO_3_ layer has been fully depleted. It has been shown that the existence and evolution of a depletion layer in a ferroelectric film will cause shrinking and tilting of its *P-E* loop, leading to reduced *P_r_* and *E_c_* [25]. On the other hand, after the BaTiO_3_ layer has been completely depleted, a substantial amount of bound charges appear at the BiFeO_3_/BaTiO_3_ interface owing to the large difference in polarizations between the two layers. Hence the charge transport of the bi-layer film under a high electric field will be dominated by the interface charge coupling, which can be described by an added energy term $\frac{1}{2\varepsilon_{0}}$*α*(1 − *α*)($\vec{P_{1}}$ − $\vec{P_{2}}$)^2^ in the Landau-Ginzburg-Devonshire (LGD) thermodynamic potential (free energy density F) of the bilayer thick film [26]:

(4) $$F=(1-\alpha {)[F}_{1}(\vec{P_{1}})-\vec{E}\cdot \vec{P_{1}}]+\alpha {[F}_{2}(\vec{P_{2}})-\vec{E}\cdot \vec{P_{2}}]+\frac{1}{2\varepsilon_{0}}\alpha (1-\alpha ){\left(\vec{P_{1}}-\vec{P_{2}}\right)}^{2}$$

here *α* = *h*_2_*/h* (*h* = *h*_1_ + *h*_2_) is the relative thickness of the second layer, $F_{i}$, $\vec{P_{i}}$ (i = 1, 2) are the bulk free energy density and polarization of the ith layer, and $\vec{E}$ is the applied electric field (see Figure 4b). The third term of the free energy density with the coefficient *α*(1 − *α*) expresses the energy of electrostatic interaction between the two layers, and it becomes dominant when the field is large enough to allow both layers become fully poled. In this case, the difference between the two polarizations diminishes and the film shows an “average” polarization. In our case, the polarization of the fully poled bi-layer film *P_bi_* is close to that of BiFeO_3_ due to the fact that *P_BFO_* >> *P_BTO_*. The computed *P_bi_* by solving the equilibrium state of Equation (4) is close to 50 μC/cm^2^ [21], fairly consistent with the observed *P_m_* value if the linear contribution from dielectric susceptibility to the total polarization is deducted. Therefore, the enhanced energy storage capability of the BiFeO_3_/BaTiO_3_ bi-layer film can be attributed to a combination of space charge effect (dominant at low field, leading to small *P_r_* and *E_c_*) and effect of interlayer charge coupling (dominant at high field, leading to a large *P_m_*).

**Figure 5:**
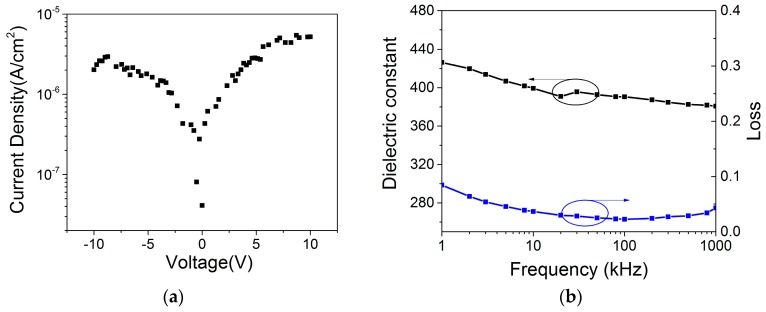
(**a**) Leakage current density vs. voltage *(J-V*) curve; and (**b**) frequency dependent dielectric properties of the BiFeO_3_/BaTiO_3_ bi-layer film.

Figure 5 a shows the leakage current density versus electric field (*J-E*) curve. At an electric field of 100 kV/cm (bias voltage of 10 V), the leakage current density of the bi-layer film is 5.0 × 10^−6^ A/cm^2^. This is about an order of magnitude lower than those reported for single layer BiFeO_3_ films grown on Si substrates [27,28]. The reduction in leakage current is in good agreement with the observed morphology change shown in AFM and SEM images, i.e., a densely and smoothly grown BiFeO_3_ film was endowed with a much improved electrical resistivity [29]. In addition, an interface energy barrier *φ*_B_ between the BiFeO_3_ (work function W ~ 4.7 eV) and BaTiO_3_ (W ~ 4.0 eV) layers can also reduce the leakage current and hence allow the film to be exposed to a large electric field [30,31]. As a result, a high energy density is achieved in the BiFeO_3_/BaTiO_3_ bi-layer film.

Figure 5b displays frequency-dependent relative dielectric constant *ε_r_* and loss tangent tan*δ* of the bi-layer film. It can be seen that the pseudo static *ε_r_* is about 425 (@1 kHz), which is about 33% and 70% higher than those of the single layer BaTiO_3_ and BFO films grown on Si, respectively (*ε_r_* ~ 320 for BTO, *ε_r_* ~ 250 for BFO) [22,32]. When the frequency increases from 1 kHz to 1 MHz, *ε_r_* decreases from ~425 to ~380. On the other hand, tan *δ* varies between 2.4% and 8.4% in the same frequency range, similar to that of pure BTO films grown on Si. The enhanced dielectric constant *ε_r_* contributes to the improved maximum polarization *P_m_,* based on the relation *P_m_* = *D_m_* = *P* + *ε_r_* × *E_max_*, where P is the self-polarization and *P* ≈ *P_r_* for ferroelectrics.

## 4. Conclusions

In this study, BiFeO_3_/BaTiO_3_ bi-layer thick films deposited on the LaNiO_3_ -buffered Pt/Ti/SiO_2_/(100) Si substrates display an enhanced energy density and charge-discharge efficiency (*W_c_* = 71 J/cm^3^, *η* = 61%) as compared with those of the single layer films. This enhancement can be attributed to combined effects of space charges and the interlayer charge coupling. The bi-layer film exhibits a low leakage current density (5.0 × 10^−6^ A/cm^2^ at 100 kV/cm) as a result of the dense and smooth film morphology achieved in the BFO layer, which is induced by the high quality underlayer of BaTiO_3_/LaNiO_3_. The relative dielectric constant *ε_r_* is about 33% higher than that of the pure BaTiO_3_ film and 70% higher than that of the pure BFO film. In conclusion, the BiFeO_3_/BaTiO_3_ bi-layer film shows excellent dielectric performance and energy storage characteristics, making this structure a promising candidate for applications in microelectronics as lead-free thin film ceramic capacitors.

## Figures and Tables

**Table 1:** Deposition Parameters of the Sputtering Process for BiFeO_3_/BaTiO_3_ Bi-layer Films on SiO_2_/(100) Si Substrates.

Sputtering Parameters	BFO	BTO	LNO
Sputtering power (W)	100		
Substrate temperature (°C)	500		
Sputtering pressure (Pa)	1.2		0.3
Sputtering atmosphere	Ar + O_2_ (4:1 flow ratio)		
Cooling atmosphere and pressure	Pure oxygen, 2.5 Pa		
Pt/Ti sputtering parameters	300 °C, 0.3 Pa, 55 W, pure Ar		

## Notes

For a ferroelectric, its *P-E* curve is close to *D-E* curve and the two notations (*P-E*, *D-E*) are often interchangeably used for one another. Rigorously, what is measured in a polarization-electric field test (*P-E* test) of a ferroelectric material is its *D-E* curve.

## References

[1] Wang Y., Zhou X., Chen Q., Chu B., Zhang Q. (2010). Recent development of high energy density polymers for dielectric capacitors. IEEE Trans. Dielectr. Electr. Insul..

[2] Hao X., Wang Y., Yang J., An S., Xu J. (2012). High energy-storage performance in Pb_0.91_La_0.09_(Ti_0.65_Zr_0.35_) O_3_ relaxor ferroelectric thin films. J. Appl. Phys..

[3] Hao X., Wang Y., Zhang L., Zhang L., An S. (2013). Composition-dependent dielectric and energy-storage properties of (Pb,La)(Zr,Sn,Ti) O_3_ antiferroelectric thick films. Appl. Phys. Lett..

[4] Zhang L., Hao X., Zhang L. (2014). Enhanced energy-storage performances of Bi_2_O_3_–Li_2_O added (1 − *x*)(Na_0.5_Bi_0.5_) TiO_3_–*x*BaTiO_3_ thick films. Ceram. Int..

[5] Xie Z., Yue Z., Ruehl G., Peng B., Zhang J., Yu Q., Zhang X., Li L. (2014). Bi(Ni_1/2_Zr_1/2_)O_3_-PbTiO_3_ relaxor-ferroelectric films for piezoelectric energy harvesting and electrostatic storage. Appl. Phys. Lett..

[6] Xie Z., Peng B., Meng S., Zhou Y., Yue Z. (2013). High–Energy-Storage Density Capacitors of Bi (Ni_1/2_Ti_1/2_) O_3_–PbTiO_3_ Thin Films with Good Temperature Stability. J. Am. Ceram. Soc..

[7] Xie Z., Peng B., Zhang J., Zhang X., Yue Z., Li L., Trolier-McKinstry S.E. (2015). Highly (100)-Oriented Bi(Ni_1/2_Hf_1/2_)O_3_-PbTiO_3_Relaxor-Ferroelectric Films for Integrated Piezoelectric Energy Harvesting and Storage System. J. Am. Ceram. Soc..

[8] Xie Z., Yue Z., Peng B., Zhang J., Zhao C., Zhang X., Ruehl G., Li L. (2015). Large enhancement of the recoverable energy storage density and piezoelectric response in relaxor-ferroelectric capacitors by utilizing the seeding layers engineering. Appl. Phys. Lett..

[9] Xie Z., Peng B., Zhang J., Zhang X., Yue Z., Li L. (2015). Effects of thermal anneal temperature on electrical properties and energy-storage density of Bi(Ni_1/2_Ti_1/2_)O_3_–PbTiO_3_ thin films. Ceram. Int..

[10] Lee B.D., Lee H.R., Yoon K.H., Cho Y.S. (2005). Microwave dielectric properties of magnesium calcium titanate thin films. Ceram. Int..

[11] Huang C.L., Chen Y.B. (2006). Structure and electrical characteristics of RF magnetron sputtered MgTiO_3_. Surf. Coat. Technol..

[12] Tkach A., Almeida A., Moreira J.A., Perez de la Cruz J., Romaguera-Barcelay Y., Vilarinho P.M. (2012). Low-temperature dielectric response of NaTaO_3_ ceramics and films. Appl. Phys. Lett..

[13] Liu G.Z., Wang C., Wang C.C., Qiu J., He M., Xing J., Jin K.J., Lu H.B., Yang G.Z. (2008). Effects of interfacial polarization on the dielectric properties of BiFeO_3_ thin film capacitors. Appl. Phys. Lett..

[14] Lee S.J., Kang K.Y., Kim J.W., Han S.K., Jeong S.D. Low-Frequency Dielectric Properties of Sol-Gel Derived BaTiO_3_ Thin Films. Proceedings of the Materials Research Society.

[15] Yuan M., Zhang W., Wang X., Pan W., Wang L., Ouyang J. (2013). In situ preparation of high dielectric constant, low-loss ferroelectric BaTiO_3_ films on Si at 500 °C. Appl. Surf. Sci..

[16] Zhu H., Sun X., Kang L., Zhang Y., Yu Z., Ouyang J., Pan W. (2016). Microstructural and electrical characteristics of epitaxial BiFeO_3_ thick films sputtered at different Ar/O_2_ flow ratios. CrystEngComm.

[17] Gao Y., Yuan M., Sun X., Ouyang J. (2016). In situ preparation of high quality BaTiO_3_ dielectric films on Si at 350–500 °C. J. Mater. Sci. Mater. Electron..

[18] Lee C.C., Wu J.M. (2007). Effect of film thickness on interface and electric properties of BiFeO_3_ thin films. Appl. Surf. Sci..

[19] Yan F., Zhu T.J., Lai M.O., Lu L. (2010). Influence of oxygen pressure on the ferroelectric properties of BiFeO_3_ thin films on LaNiO_3_/Si substrates via laser ablation. Appl. Phys. A.

[20] Dho J., Qi X., Kim H., MacManus-Driscoll J.L., Blamire M.G. (2006). Large Electric Polarization and Exchange Bias in Multiferroic BiFeO_3_. Adv. Mater..

[21] Zhu H., Liu M., Zhang Y., Yu Z., Ouyang J., Pan W. (2016). Increasing energy storage capabilities of space-charge dominated ferroelectric thin films using interlayer coupling. Acta Mater..

[22] Hussain S., Hasanain S.K., Hassnain Jaffari G., Ismat Shah S. (2015). Thickness dependent magnetic and ferroelectric properties of LaNiO_3_ buffered BiFeO_3_ thin films. Curr. Appl. Phys..

[23] Yuan M.L. (2014). Medium Temperature Preparation of BaTiO_3_ Thin Film Capacitors with High Breakdown Voltages and Large Electric Energy Densities. Master’s Thesis.

[24] Zhang W., Gao Y., Kang L., Yuan M., Yang Q., Cheng H., Pan W., Ouyang J. (2015). Space-charge dominated epitaxial BaTiO_3_ heterostructures. Acta Mater..

[25] Tagantsev A.K., Landivar M., Colla E., Setter N. (1995). Identification of passive layer in ferroelectric thin films from their switching parameters. J. Appl. Phys..

[26] Roytburd A.L., Zhong S., Alpay S.P. (2005). Dielectric anomaly due to electrostatic coupling in ferroelectric-paraelectric bilayers and multilayers. Appl. Phys. Lett..

[27] Liu Y.T., Ku C.S., Chiu S.J., Lee H.Y., Chen S.Y. (2014). Ultrathin oriented BiFeO_3_ films from deposition of atomic layers with greatly improved leakage and ferroelectric properties. ACS Appl. Mater. Interfaces.

[28] Simões A.Z., Riccardi C.S., Dos Santos M.L., Garcia F.G., Longo E., Varela J.A. (2009). Effect of annealing atmosphere on phase formation and electrical characteristics of bismuth ferrite thin films. Mater. Res. Bull..

[29] Kawae T., Terauchi Y., Tsuda H., Kumeda M., Morimoto A. (2009). Improved leakage and ferroelectric properties of Mn and Ti codoped BiFeO_3_ thin films. Appl. Phys. Lett..

[30] Wang C., Jin K.J., Xu Z.T., Wang L., Ge C., Lu H.B., Guo H.Z., He M., Yang G.Z. (2011). Switchable diode effect and ferroelectric resistive switching in epitaxial BiFeO_3_ thin films. Appl. Phys. Lett..

[31] Sun S., Wang Y., Fuierer P.A., Tuttle B.A. (1999). Annealing effects on the internal bias field in ferroelectric PZT thin films with self-polarization. Integr. Ferroelectr..

[32] Yan F., Zhu T.J., Lai M.O., Lu L. (2011). Effect of bottom electrodes on nanoscale switching characteristics and piezoelectric response in polycrystalline BiFeO_3_ thin films. J. Appl. Phys..

